# Supplementary material for: Focus image scanning microscopy for sharp and gentle super-resolved microscopy
Source: Nat Commun. 2022 Dec 13;13:7723. doi: 10.1038/s41467-022-35333-y (PMC9747786; doi:10.1038/s41467-022-35333-y)
Supplement: Supplementary file 1 — Supplementary Information [file 41467_2022_35333_MOESM1_ESM.pdf]

Focus Image Scanning Microscopy for Sharp and Gentle Super-Resolved Microscopy

Supplementary Material

Giorgio Tortarolo[1,†,♯], Alessandro Zunino[1,†], Francesco Fersini[1,2], Marco Castello[1,3], Simonluca Piazza[1,3], Colin J.R. Sheppard[1,3], Paolo Bianchini[3], Alberto Diaspro[3,4], Sami Koho[1], Giuseppe Vicidomini[1,*]

[1]Molecular Microscopy and Spectroscopy, Istituto Italiano di Tecnologia, Via Enrico Melen, 83, 16152, Genoa, Italy

[2]Dipartimento di Informatiche, Bioingegneria, Robotica e Ingegneria dei Sistemi, University of Genoa, Via All'Opera Pia 13, 16145, Genoa, Italy

[3]Nanoscopy and NIC@IIT, Istituto Italiano di Tecnologia, Via Enrico Melen, 83, 16152, Genoa, Italy

[4]Dipartimento di Fisica, University of Genoa, Via Dodecaneso, 33, 16146, Genoa, Italy

[*] Corresponding authors: giuseppe.vicidomini@iit.it.

[†] These authors contributed equally to this work.

[♯] Current affiliation: Laboratory of Experimental Biophysics, Ecole Polytechnique Fderale de Lausanne, Rte Cantonale, 1015, Lausanne, Switzerland

1 Adaptive Pixel Reassignment

From a statistical point of view, the pixel-reassignment (PR) concept reassigns the micro-image's photons $i(\mathbf{x}_d|\mathbf{x}_s)$ to the most likely position of origin in the sample space $\mathbf{x}$ (Fig. 1b). For a given scanning position $\mathbf{x}_s$, the most likely point from which the photons detected at the position $\mathbf{x}_d$ originate is the maximum point (mode) of the joint probability, or effective point-spread function, $h = h_{\text{exc}} \times h_{\text{det}}$. Here h_{exc} is the excitation PSF, i.e., the probability to excite a fluorophore as a function of its position, and h_{det} is the detection PSF, i.e.., the probability of detecting a photon – at $\mathbf{x}_d$ – as a function of the fluorophore position. If we denote with $\boldsymbol{\mu}'(\mathbf{x}_d)$ the displacement between the detection position $\mathbf{x}_d$ and the effective PSF maximum – known as the micro-image shift-vector, PR consists in repositioning the photon location from $\mathbf{x}_d$ to $\mathbf{x} = \mathbf{x}_s - \mathbf{x}_d + \boldsymbol{\mu}'(\mathbf{x}_d)$. Notably, if $\boldsymbol{\mu}'(\mathbf{x}_d)$ is null, the resulting PR image is the conventional wide-field image. Instead, if $\boldsymbol{\mu}'(\mathbf{x}_d) = \mathbf{x}_d$ then all photons are assigned to the scanning position $\mathbf{x}_s$ and the PR image is the conventional LSM image (with an open pinhole) [1].Given the micro-images and the relative shift-vectors, the PR operation can be easily implemented computationally.

To avoid post-processing, different opto-mechanical PR implementations have been proposed, assuming no Stokes-shift, and Gaussian shaped excitation and detection PSFs. Under these assumptions, the maximum of the joint probability is located at the middle of the detection and excitation PSFs' positions, namely $\boldsymbol{\mu}'(\mathbf{x}_d) = \mathbf{x}_d/2$. Mechanical ISM [2, 3] implements PR by re-scanning the fluorescent signal with twice larger scan amplitude – than used by scanning the excitation beam. Optical ISM [4, 5] adopts a multi-spot excitation scheme and implements the PR by demagnifying twice the fluorescent spots. Interestingly, some computational ISM implementations [6, 7] also use the multi-spots scheme to speed-up imaging rate.

Practically, however, the Stokes-shift is not negligible, and the excitation and detection PSFs may deviate from ideal Gaussian shapes. These aspects reduce the versatility and robustness of the opto-mechanical ISM implementations. Computational ISM implementations require strategies to effectively determine the micro-image shift-vector $\boldsymbol{\mu}'(\mathbf{x}_d)$. In this context, we have introduced the adaptive pixel-reassignment (APR) method, which automatically adapts to the Stokes-shift and to realistic PSF shapes. To explain the APR method, it is necessary to regard the raw ISM dataset from a different point of view: each element of the detector array can be considered as an independent detector generating a confocal image with an offset pinhole, the so-called scanned-image. In other words, the raw ISM dataset can be regarded both as a collection of micro-images $i(\mathbf{x}_d|\mathbf{x}_s)$ – one for each sample position $\mathbf{x}_s$, or a collection of scanned-images $i(\mathbf{x}_s|\mathbf{x}_d)$ – one for each detector position $\mathbf{x}_d$. From the latter point of view, the PR method reassigns the scanned-image's photons $i(\mathbf{x}_s|\mathbf{x}_d)$ to the most likely position of origin in the sample space $\mathbf{x}$. Because this position is encoded in the scanned-image, we obtain the scanned-image shift-vector $\boldsymbol{\mu}(\mathbf{x}_d)$ by extracting the relative displacements between the scanned-images. Thus, the new position of the photons reads $\mathbf{x} = \mathbf{x}_s - \boldsymbol{\mu}(\mathbf{x}_d)$.

Notably, given the scanned-image shift-vector one can derive the micro-image shift-vector, i.e., $\boldsymbol{\mu}'(\mathbf{x}_d) = \mathbf{x}_d - \boldsymbol{\mu}(\mathbf{x}_d)$. The APR is effectively implemented by shifting the scanned images by $\boldsymbol{\mu}(\mathbf{x}_d)$ and summing them up.

2 Working principle of Focus-ISM

The point-spread function (PSF) of the scanned-image generated by the element at position $\mathbf{x}_d$ of a detector array is given by the PSF of a laser scanning microscope, just with a shifted pinhole aperture [8, 9]. The equation is

$$h(\mathbf{x}_s|\mathbf{x}_d) = h_{\text{exc}}(-\mathbf{x}_s) \cdot [h_{\text{det}}(\mathbf{x}_s) * p(\mathbf{x}_s - \mathbf{x}_d)] \tag{S1}$$

where $\mathbf{x}_s = (x_s, y_s)$ are the scan coordinates on the sample plane in focus, p is the pinhole aperture that describes the sensitive area of the element of the detector array located at $\mathbf{x}_d = (x_d, y_d)$, and h_{exc} and h_{det} are, respectively, the excitation and detection normalized PSFs ($\int h_{\text{exc}} = \int h_{\text{det}} = 1$).

For STED-ISM, we also have to consider the reduction of the effective fluorescence region, which is equivalent to a reduction of the effective excitation PSF

$$h^{(\varsigma)}_{\text{exc}}(\mathbf{x}_s) = h^{(0)}_{\text{exc}}(\mathbf{x}_s) \cdot \eta[\varsigma(\mathbf{x}_s)] \tag{S2}$$

where $h^{(0)}_{\text{exc}}$ is the confocal excitation PSF, and η is the depletion function, which describes the reduction of fluorescence emission under exposure of the depletion beam. ς is the saturation factor, defined as

$$\varsigma = I_{\text{STED}}/I_{\text{sat}} \tag{S3}$$

where $I_{\text{STED}}(\mathbf{x}_s)$ is the doughnut-shaped intensity distribution of the STED beam and I_{sat} is the saturation intensity, namely the intensity of stimulated emission needed to have spontaneous emission as likely as stimulated emission. In other words, the spontaneous emission rate k_F equals the stimulated emission rate k_{SE} when $I_{\text{STED}} = I_{\text{sat}}$. In order to calculate the saturation factor ς using a pulsed laser, we need to take into account the dynamics of the fluorescence intensity $F(t)$ under stimulated emission [8]. The fluorescence decay is

$$F(\varsigma, t) \propto \exp(-k_F t) \cdot \exp[-\varsigma k_F \min(t, T)] \tag{S4}$$

where T is the pulse duration of the STED beam. Assuming a time-gated detection in the microscope (namely, the light is collected only after the STED beam pulse is over), the depletion function for the gated pulsed STED implementation reads

$$\eta_{gp}(\varsigma) = \frac{\int_T^{+\infty} F(\varsigma, t)dt}{\int_T^{+\infty} F(0, t)dt} = \exp(-\varsigma k_F T) \tag{S5}$$

Finally, the equation for the depleted excitation PSF is

$$h^{(\varsigma)}_{\text{exc}}(\mathbf{x}_s) = h^{(0)}_{\text{exc}}(\mathbf{x}_s) \cdot \exp[-k_F T \varsigma(\mathbf{x}_s)] \tag{S6}$$

Since $\varsigma(\mathbf{x}_s)$ is doughnut-shaped, the excitation PSF is more depleted at its borders and its effective width is smaller. Notably, the STED excitation PSF is no longer normalized.

For the rest of the manuscript, we will indicate with h_{exc} a generic excitation PSF with effective width depending on the saturation factor. Thus, the scanned-image generated by the detector element at position $\mathbf{x}_d$ can be written as

$$i(\mathbf{x}_s|\mathbf{x}_d) = o(\mathbf{x}_s) * h(\mathbf{x}_s|\mathbf{x}_d) \tag{S7}$$

where $o(\mathbf{x}_s)$ is the specimen, here considered as a distribution of light emitters. In this work, we consider the limit case where each element of the SPAD array can be described as a shifted Dirac delta function. Substituting $p(\mathbf{x}_s)$ with $\delta(\mathbf{x}_s - \mathbf{x}_d)$ in equation S1 we obtain

$$h(\mathbf{x}_s|\mathbf{x}_d) = h_{\mathrm{exc}}(-\mathbf{x}_s) \cdot h_{\mathrm{det}}(\mathbf{x}_s - \mathbf{x}_d) \tag{S8}$$

In order to grasp the core idea behind image scanning microscopy (ISM), we first introduce its concept using a Gaussian approximation and later proceed with the general case.

2.1 Gaussian model

In this subsection, we simplify the model approximating the emission and excitation PSF with a Gaussian distribution with circular symmetry,

$$g(\mathbf{x}|\boldsymbol{\mu}, \sigma) = \frac{1}{2\pi\sigma^2} \exp\left[-\frac{(\mathbf{x}-\boldsymbol{\mu})^2}{2\sigma^2}\right] \tag{S9}$$

where $\boldsymbol{\mu}$ is the mean and σ is the standard deviation. Thus, the resulting PSF is

$$h(\mathbf{x}_s|\mathbf{x}_d) = g(\mathbf{x}_s|\mathbf{0}, \sigma_{\mathrm{exc}}) \cdot g(\mathbf{x}_s|\mathbf{x}_d, \sigma_{\mathrm{det}}) \tag{S10}$$

Notably, the product of two Gaussian function is another Gaussian function with the following properties:

$$\boldsymbol{\mu}(\mathbf{x}_d) = \frac{\boldsymbol{\mu}_{\mathrm{det}}\sigma_{\mathrm{exc}}^2 + \boldsymbol{\mu}_{\mathrm{exc}}\sigma_{\mathrm{det}}^2}{\sigma_{\mathrm{det}}^2 + \sigma_{\mathrm{exc}}^2} = \frac{\mathbf{x}_d\sigma_{\mathrm{exc}}^2}{\sigma_{\mathrm{det}}^2 + \sigma_{\mathrm{exc}}^2} \qquad \sigma_{\mathrm{ism}} = \sqrt{\frac{\sigma_{\mathrm{det}}^2\sigma_{\mathrm{exc}}^2}{\sigma_{\mathrm{det}}^2 + \sigma_{\mathrm{exc}}^2}} \tag{S11}$$

where $\boldsymbol{\mu}(\mathbf{x}_d)$ is known as *shift-vector*, and σ_{ism} is independent of $\mathbf{x}_d$. The intensity of the resulting Gaussian function is also rescaled by the following rescaling factor

$$s(\mathbf{x}_d) = \frac{1}{2\pi\left(\sigma_{\mathrm{det}}^2 + \sigma_{\mathrm{exc}}^2\right)} \exp\left[-\frac{\mathbf{x}_d^2}{2(\sigma_{\mathrm{det}}^2 + \sigma_{\mathrm{exc}}^2)}\right] \tag{S12}$$

Thus, the resulting PSF can be written as

$$h(\mathbf{x}_s|\mathbf{x}_d) = s(\mathbf{x}_d) \cdot g[\mathbf{x}_s|\boldsymbol{\mu}(\mathbf{x}_d), \sigma] \tag{S13}$$

Neglecting the Stokes-shift, we can assume $\sigma_{\text{exc}} \sim \sigma_{\text{det}}$ to obtain the well-known $\sqrt{2}$ gain in resolution. However, the images generated by each detector element are shifted with respect to the central element. Indeed, the scanned-image is

$$i(\mathbf{x}_s|\mathbf{x}_d) = o(\mathbf{x}_s) * h(\mathbf{x}_s|\mathbf{x}_d) = i[\mathbf{x}_s - \boldsymbol{\mu}(\mathbf{x}_d)] \tag{S14}$$

If all the images are simply summed together, the resolution gain is lost, and the resulting image is equivalent to that generated by a traditional confocal microscope with an open pinhole (as large as the detector size). The pixel reassignment (PR) algorithm shifts back each image

$$i(\mathbf{x}_s|\mathbf{x}_d) \xrightarrow[\text{PR}]{} i[\mathbf{x}_s + \boldsymbol{\mu}(\mathbf{x}_d)|\mathbf{x}_d] = i(\mathbf{x}_s|\mathbf{0}) \cdot s(\mathbf{x}_d) \tag{S15}$$

and constructs a new image summing the reassigned images

$$i_{\text{ism}}(\mathbf{x}_s) = i(\mathbf{x}_s|\mathbf{0}) \cdot \int_{\mathbb{R}^2} s(\mathbf{x}_d)\,\mathrm{d}\mathbf{x}_d \tag{S16}$$

thus conserving the gain in resolution and enhancing the signal-to-noise ratio (SNR).

2.2 General model

The typical assumption behind ISM is that the complete PSF can still be written as a new shifted function

$$h_{\text{exc}}(-\mathbf{x}_s) \cdot h_{\text{det}}(\mathbf{x}_s - \mathbf{x}_d) \propto h[\mathbf{x}_s - \boldsymbol{\mu}(\mathbf{x}_d)] \tag{S17}$$

Thus, the scanned-images generated by each detector are all of similar shape, but shifted,

$$i(\mathbf{x}_s|\mathbf{x}_d) = o(\mathbf{x}_s) * h(\mathbf{x}_s|\mathbf{x}_d) \propto i[\mathbf{x}_s - \boldsymbol{\mu}(\mathbf{x}_d)] \tag{S18}$$

Instead of using the theoretical values found using the Gaussian approximation, the shift vectors are found by the adaptive pixel reassignment (APR) algorithm. This latter first calculates the correlogram

$$R(\mathbf{x}_s|\mathbf{x}_d) = \mathcal{F}^{-1}\left\{ \frac{\mathcal{F}\{i(\mathbf{x}_s|\mathbf{x}_d)\} \cdot \overline{\mathcal{F}\{i(\mathbf{x}_s|\mathbf{0})\}}}{\left|\mathcal{F}\{i(\mathbf{x}_s|\mathbf{x}_d)\} \cdot \overline{\mathcal{F}\{i(\mathbf{x}_s|\mathbf{0})\}}\right|} \right\} \tag{S19}$$

and later finds the shift vectors as the position of maximum correlation

$$\boldsymbol{\mu}(\mathbf{x}_d) = \arg\max_{\mathbf{x}_s} \{R(\mathbf{x}_s|\mathbf{x}_d)\} \tag{S20}$$

and shifts each scanned image of the corresponding shift vector

$$i(\mathbf{x}_s|\mathbf{x}_d) \xrightarrow[\text{APR}]{} i[\mathbf{x}_s + \boldsymbol{\mu}(\mathbf{x}_d)|\mathbf{x}_d] \tag{S21}$$

with the purpose of making all the scanned-images identical, except for an intensity scale factor

$$\forall(\mathbf{x}_d, \mathbf{x}'_d) \qquad i[\mathbf{x}_s + \boldsymbol{\mu}(\mathbf{x}_d)] \propto i[\mathbf{x}_s + \boldsymbol{\mu}(\mathbf{x}'_d)] \tag{S22}$$

Rather than considering the scanned-image, the ISM image formation process can be seen equivalently from the perspective of the detector. In fact, the detector array can be considered as a small camera, capable of acquiring wide-field images that we call *micro-images.* The equation of these latter can be found as follows:

$$\begin{aligned}
i(\mathbf{x}_s|\mathbf{x}_d) &= o(\mathbf{x}_s) * [h_{\text{exc}}(-\mathbf{x}_s) \cdot h_{\text{det}}(\mathbf{x}_s - \mathbf{x}_d)] = \\
&= \int_{\mathbb{R}^2} o(\mathbf{x}_s - \mathbf{x}') \cdot h_{\text{exc}}(-\mathbf{x}') \cdot h_{\text{det}}(\mathbf{x}' - \mathbf{x}_d)\,\mathrm{d}\mathbf{x}' = \\
&= \int_{\mathbb{R}^4} o(\mathbf{x}_s - \mathbf{x}') \cdot h_{\text{exc}}(-\mathbf{x}') \cdot h_{\text{det}}(\mathbf{x}'')\delta(\mathbf{x}'' - \mathbf{x}' + \mathbf{x}_d)\,\mathrm{d}\mathbf{x}'\,\mathrm{d}\mathbf{x}'' = \\
&= \int_{\mathbb{R}^2} o(\mathbf{x}_s - \mathbf{x}'' - \mathbf{x}_d) \cdot h_{\text{exc}}(-\mathbf{x}'' - \mathbf{x}_d) h_{\text{det}}(\mathbf{x}'')\,\mathrm{d}\mathbf{x}'' = \\
&= [o(\mathbf{x}_s - \mathbf{x}_d) \cdot h_{\text{exc}}(-\mathbf{x}_d)] * h_{\text{det}}(-\mathbf{x}_d) = i(\mathbf{x}_d|\mathbf{x}_s)
\end{aligned} \tag{S23}$$

As expected, the micro-image is simply given by the wide-field image formation law, but applied to the illuminated object, i.e. the object multiplied by the excitation PSF. After APR, the reassigned micro-image is

$$i[\mathbf{x}_d|\mathbf{x}_s + \boldsymbol{\mu}(\mathbf{x}_d)] = \{o[\mathbf{x}_s - \mathbf{x}_d + \boldsymbol{\mu}(\mathbf{x}_d)] \cdot h_{\text{exc}}(-\mathbf{x}_d)\} * h_{\text{det}}(-\mathbf{x}_d) \tag{S24}$$

For clarity, we now rewrite the new coordinates of the micro-images as

$$\mathbf{u} = \mathbf{x}_d - \boldsymbol{\mu}(\mathbf{x}_d) = \mathbf{x}_d \frac{\sigma_{\text{det}}^2}{\sigma_{\text{det}}^2 + \sigma_{\text{exc}}^2} \tag{S25}$$

where we have used the theoretical value of the shift-vectors found using the Gaussian approximation for simplicity. In this new coordinate system, the post-APR micro-image is

$$i(\mathbf{u}|\mathbf{x}_s) = \left[o(\mathbf{x}_s - \mathbf{u}) \cdot h_{\text{exc}}\left(-\frac{\sigma_{\text{det}}^2 + \sigma_{\text{exc}}^2}{\sigma_{\text{det}}^2}\mathbf{u}\right)\right] * h_{\text{det}}\left(-\frac{\sigma_{\text{det}}^2 + \sigma_{\text{exc}}^2}{\sigma_{\text{det}}^2}\mathbf{u}\right) \tag{S26}$$

In other words, the effect of APR is a digital shrinking of the excitation and detection PSFs with respect to the object. In detail, the standard deviations of the PSFs become

$$\sigma_{\text{exc}} \xrightarrow[\text{APR}]{} \sigma_{\text{exc}} \frac{\sigma_{\text{det}}^2}{\sigma_{\text{det}}^2 + \sigma_{\text{exc}}^2} \qquad\qquad \sigma_{\text{det}} \xrightarrow[\text{APR}]{} \sigma_{\text{det}} \frac{\sigma_{\text{det}}^2}{\sigma_{\text{det}}^2 + \sigma_{\text{exc}}^2} \tag{S27}$$

which are always smaller than the original standard deviation.

The relation S22 applies also to the micro-images

$$\forall(\mathbf{x}_d, \mathbf{x}'_d) \qquad i[\mathbf{x}_d|\mathbf{x}_s + \boldsymbol{\mu}(\mathbf{x}_d)] \propto i[\mathbf{x}_d|\mathbf{x}_s + \boldsymbol{\mu}(\mathbf{x}'_d)] \tag{S28}$$

Notably, the proportionality factor in the above relation is independent of $\mathbf{x}_s$. Thus, the above condition is satisfied only if equation S24 can be factored into a term depending only on $\mathbf{x}_s$ and another term depending only on $\mathbf{x}_d$. This can occur only in the following cases:

1. If o is constant over the whole space, namely it is a flat object. This case is trivial and we will not consider it.

2. If $\boldsymbol{\mu}(\mathbf{x}_d) = \mathbf{x}_d$, then o depends only on $\mathbf{x}_s$ and is constant with respect to $\mathbf{x}_d$. This case is satisfied only if $h_{\text{exc}} = \text{const}$, namely using a wide-field illumination. This would come at the cost of sacrificing resolution and it is not of our interest.

3. If it is possible to achieve a perfect pixel reassignment. This case is possible only if we can rewrite the product $h_{\text{exc}}(-\mathbf{x}_s) \cdot h_{\text{det}}(\mathbf{x}_s - \mathbf{x}_d)$ exactly as a new shifted function $h(\mathbf{x}_s - \boldsymbol{\mu})$. Namely, the shape of the complete PSF h has to be independent of $\mathbf{x}_d$. This condition can be satisfied in ideal confocal microscopy.

4. If the illumination spot is so small that only the position $\mathbf{x}_s$ of the object o contributes to image formation. This case is satisfied only using a point-like excitation $h_{\text{exc}}(\mathbf{x}_d) = \delta(\mathbf{x}_d)$, as in ideal STED microscopy.

We are interested only in the third and fourth case, analyzed in detail in the following sections.

2.3 The confocal case

In this section, we assume that the following relation is exact

$$h_{\text{exc}}(-\mathbf{x}_s) \cdot h_{\text{det}}(\mathbf{x}_s - \mathbf{x}_d) = h[\mathbf{x}_s - \boldsymbol{\mu}(\mathbf{x}_d)] \tag{S29}$$

where the complete PSF $h[\mathbf{x}_s - \boldsymbol{\mu}(\mathbf{x}_d)]$ in general is not normalized. The normalization factor is calculated as

$$\begin{aligned} s(\mathbf{x}_d) &= \int_{\mathbb{R}^2} h[\mathbf{x}_s - \boldsymbol{\mu}(\mathbf{x}_d)]\, \mathrm{d}\mathbf{x}_s = \int_{\mathbb{R}^2} h_{\text{exc}}(-\mathbf{x}_s) \cdot h_{\text{det}}(\mathbf{x}_s - \mathbf{x}_d)\, \mathrm{d}\mathbf{x}_s = \\ &= h_{\text{exc}}(-\mathbf{x}_d) * h_{\text{det}}(-\mathbf{x}_d) \end{aligned} \tag{S30}$$

Thus, we can rewrite the PSF as

$$h[\mathbf{x}_s - \boldsymbol{\mu}(\mathbf{x}_d)] = s(\mathbf{x}_d) \cdot g[\mathbf{x}_s - \boldsymbol{\mu}(\mathbf{x}_d)] \tag{S31}$$

where $g(\mathbf{x}_s)$ is a normalized function. Using assumption S29, we replicate the calculations that led to equation S23 after performing APR

$$
\begin{aligned}
i(\mathbf{x}_s + \boldsymbol{\mu}(\mathbf{x}_d)|\mathbf{x}_d) &= o[\mathbf{x}_s + \boldsymbol{\mu}(\mathbf{x}_d)] * h[\mathbf{x}_s - \boldsymbol{\mu}(\mathbf{x}_d)] = \\
&= \{o[\mathbf{x}_s + \boldsymbol{\mu}(\mathbf{x}_d)] * g[\mathbf{x}_s - \boldsymbol{\mu}(\mathbf{x}_d)]\} \cdot s(\mathbf{x}_d) = \\
&= [o(\mathbf{x}_s) * g(\mathbf{x}_s)] \cdot s(\mathbf{x}_d) = i(\mathbf{x}_d|\mathbf{x}_s + \boldsymbol{\mu}(\mathbf{x}_d))
\end{aligned}
\tag{S32}
$$

Notably, the scaling factor $s(\mathbf{x}_d)$ is exactly the convolution of the excitation PSF with the detection PSF. Thus, we can write the equation of the micro-image after APR as

$$
i(\mathbf{x}_d) = \alpha \cdot [h_{\text{exc}}(-\mathbf{x}_d) * h_{\text{det}}(-\mathbf{x}_d)]
\tag{S33}
$$

where $\alpha = o(\mathbf{x}_s) * g(\mathbf{x}_s)$ is a proportionality factor with the units of a photon flux. Thus, the reassigned micro-image equals the *fingerprint* of the image, except for an intensity scale factor. The *fingerprint* $f(\mathbf{x}_d)$ of the system is defined as the integral of the image $i(\mathbf{x}_s|\mathbf{x}_d)$ over all the scan points $\mathbf{x}_s$

$$
\begin{aligned}
f(\mathbf{x}_d) &= \int_{\mathbb{R}^2} i(\mathbf{x}_s|\mathbf{x}_d)\,\mathrm{d}^2\mathbf{x}_s = \int_{\mathbb{R}^2} o(\mathbf{x}_s) * [h_{\text{exc}}(-\mathbf{x}_s) \cdot h_{\text{det}}(\mathbf{x}_s - \mathbf{x}_d)]\,\mathrm{d}\mathbf{x}_s = \\
&= \int_{\mathbb{R}^2} o(\mathbf{x}_s)\,\mathrm{d}\mathbf{x}_s \int_{\mathbb{R}^2} h_{\text{exc}}(-\mathbf{x}_s) \cdot h_{\text{det}}(\mathbf{x}_s - \mathbf{x}_d)\,\mathrm{d}\mathbf{x}_s = \\
&= \gamma \cdot [h_{\text{exc}}(-\mathbf{x}_d) * h_{\text{det}}(-\mathbf{x}_d)]
\end{aligned}
\tag{S34}
$$

where $\gamma = \int_{\mathbb{R}^2} o(\mathbf{x}_s)\,\mathrm{d}\mathbf{x}_s$ is a scale factor that depends only on the sample distribution.

Therefore, we have found that under the assumption S29, the reassigned micro-image is proportional to the fingerprint. Importantly, this mathematical relation is satisfied by a Gaussian PSF, which is well known to be a good approximation for both detection and excitation PSFs if $\|\mathbf{x}_d\| \lesssim 1$ Airy Unit. However, our result is more general, being valid for each function that satisfies equation S29. Moreover, APR is an adaptive algorithm which can compensate for mild deviations from ideality, since the shift-vectors are calculated as the translations that maximize the similarity between the scanned images.

2.4 The STED case

Assuming an ideal STED system, we can impose a point-like excitation

$$
h_{\text{exc}}(\mathbf{x}_d, z_0) \underset{\varsigma \to \infty}{\sim} \varepsilon \cdot \delta(\mathbf{x}_d)
\tag{S35}
$$

where ε is a (theoretically vanishing) scale factor. In this case, the shift vectors are null

$$
\boldsymbol{\mu}(\mathbf{x}_d) = \mathbf{x}_d \frac{\sigma^2_{\text{exc}}}{\sigma^2_{\text{det}} + \sigma^2_{\text{exc}}} \xrightarrow[\sigma_{\text{exc}} \to 0]{} \mathbf{0}
\tag{S36}
$$

Thus, APR does not modify the micro-images. Using assumption S35, equation S23 becomes

(S37)
$$i(\mathbf{x}_d|\mathbf{x}_s) = \alpha \cdot h_{\text{det}}(-\mathbf{x}_d)$$

where $\alpha = \varepsilon \cdot o(\mathbf{x}_s)$ is a proportionality factor with the units of a photon flux. We found the interesting result that the micro-image obtained while performing STED microscopy is the detection PSF.

Applying condition S35 to equation S34, we obtain the fingerprint of a STED image

(S38)
$$f(\mathbf{x}_d) = \gamma \cdot h_{\text{det}}(-\mathbf{x}_d)$$

which corresponds to the detection PSF, except for a scale factor.

2.5 Focus-ISM

We have found that, after APR and under reasonable assumptions, the micro-images of an ISM dataset are proportional to the fingerprint of the image. In this section, we generalize the equations for the micro-images and for the fingerprint to the case of a three-dimensional object $o(\mathbf{x}_s, z)$. The micro-image of a 3D object is

(S39)
$$i(\mathbf{x}_d|\mathbf{x}_s, z) = \int_{\mathbb{R}} [o(\mathbf{x}_s - \mathbf{x}_d, z') \cdot h_{\text{exc}}(-\mathbf{x}_d, z - z')] * h_{\text{det}}(-\mathbf{x}_d, z - z')\, \mathrm{d}z'$$

where the convolution is calculated along the dimensions (x_d, y_d). Assuming that the sample is localized on a single plane, we have $o(\mathbf{x}_s - \mathbf{x}_d, z) = o(\mathbf{x}_s - \mathbf{x}_d)\delta(z - z_0)$. Using this assumption, we obtain the micro-image of an object o placed at distance z_0 from the image plane z

(S40)
$$i(\mathbf{x}_d|\mathbf{x}_s, z) = [o(\mathbf{x}_s - \mathbf{x}_d, z_0) \cdot h_{\text{exc}}(-\mathbf{x}_d, z - z_0)] * h_{\text{det}}(-\mathbf{x}_d, z - z_0)$$

After pixel reassignment, the micro-image becomes

(S41)
$$i(\mathbf{x}_d|\mathbf{x}_s, z) = \alpha(\mathbf{x}_s, z_0) \cdot [h_{\text{exc}}(-\mathbf{x}_d, z - z_0)] * h_{\text{det}}(-\mathbf{x}_d, z - z_0)]$$

The *fingerprint* of an image generated by a 3D distribution of emitters $o(\mathbf{x}_s, z)$ is

(S42)
$$\begin{aligned} f(\mathbf{x}_d, z) &= \int_{\mathbb{R}^2} i(\mathbf{x}_s|\mathbf{x}_d)\, \mathrm{d}\mathbf{x}_s = \int_{\mathbb{R}^2} o(\mathbf{x}_s, z) * h(\mathbf{x}_s, z|\mathbf{x}_d)\, \mathrm{d}\mathbf{x}_s = \\ &= \int_{\mathbb{R}^2} o(\mathbf{x}_s, z) * [h_{\text{exc}}(-\mathbf{x}_s, z) \cdot h_{\text{det}}(\mathbf{x}_s - \mathbf{x}_d, z)]\, \mathrm{d}\mathbf{x}_s = \\ &= \int_{\mathbb{R}} \left[\int_{\mathbb{R}^2} o(\mathbf{x}_s, z - z')\, \mathrm{d}\mathbf{x}_s \int_{\mathbb{R}^2} h_{\text{exc}}(-\mathbf{x}_s, z') \cdot h_{\text{det}}(\mathbf{x}_s - \mathbf{x}_d, z')\, \mathrm{d}\mathbf{x}_s \right] \mathrm{d}z' = \\ &= \int_{\mathbb{R}} \gamma(z - z') \cdot [h_{\text{exc}}(-\mathbf{x}_d, z') * h_{\text{det}}(-\mathbf{x}_d, z')\, \mathrm{d}\mathbf{x}_s]\, \mathrm{d}z' = \\ &= \gamma(z) * [h_{\text{exc}}(-\mathbf{x}_d, z) * h_{\text{det}}(-\mathbf{x}_d, z)] \end{aligned}$$

where $\gamma(z) = \int_{\mathbb{R}^2} o(\mathbf{x}_s, z)\, \mathrm{d}\mathbf{x}_s$ is a weight function that depends only on the sample distribution, and the first convolution is calculated over the z-axis, while the second convolution is calculated over the $\mathbf{x}_s$ axes. Using the assumption that the sample is localised on a single plane, we have $\gamma(z) = \gamma_0 \delta(z - z_0)$ and we obtain

$$f(\mathbf{x}_d, z - z_0) = \gamma_0 \cdot h_{\mathrm{exc}}(-\mathbf{x}_d, z - z_0) * h_{\mathrm{det}}(-\mathbf{x}_d, z - z_0) \tag{S43}$$

which is proportional to equation S41. We can use this result to write the equation of a micro-image generated by multiple emitters at different axial planes

$$i(\mathbf{x}_d) = \sum_{k=1}^{K} \alpha_k \cdot f(\mathbf{x}_d, z_k) \tag{S44}$$

Notably the fingerprint is always centered with respect to the detector array, but its lateral width depends on the axial position of the emitter. Approximating again each PSF with a Gaussian function, the fingerprint is a Gaussian function as well. Considering only two contributes (in-focus and out-of-focus), we finally obtain

$$i(\mathbf{x}_d) = \alpha \cdot g(\mathbf{x}_d | \mathbf{0}, \sigma_{\mathrm{sig}}) + \beta \cdot g(\mathbf{x}_d | \mathbf{0}, \sigma_{\mathrm{bkg}}) \tag{S45}$$

where the weights of the Gaussian functions respect the conservation of the energy constraint

$$\alpha + \beta = \int_{\Sigma} i(\mathbf{x}_d)\, \mathrm{d}^2\mathbf{x}_d \tag{S46}$$

Notably, the standard deviation of the in-focus component can easily be measured experimentally. The fingerprint is effectively a micro-image averaged over many scan points. Thus, the SNR of the fingerprint is much higher than that of a micro-image. We can exploit this information to calibrate σ_{sig}. This process can be performed with a separate experiment, or alternatively, it is possible to calculate the *fingerprint* on a region of $i(\mathbf{x}_s|\mathbf{x}_d)$ containing only in-focus emitters. If fitted to a Gaussian function, the fingerprint of the sub-image provides an experimental measure of σ_{sig}.

Once each micro-image has been separated in an in-focus and out-of-focus component, the corresponding images are constructed as follows

$$i_{\mathrm{sig}}(\mathbf{x}_s) = \alpha(\mathbf{x}_s) \cdot \int_{\mathbb{R}^2} g(\mathbf{x}_d | \mathbf{0}, \sigma_{\mathrm{sig}})\, \mathrm{d}\mathbf{x}_d \tag{S47}$$

$$i_{\mathrm{bkg}}(\mathbf{x}_s) = \beta(\mathbf{x}_s) \cdot \int_{\mathbb{R}^2} g(\mathbf{x}_d | \mathbf{0}, \sigma_{\mathrm{bkg}})\, \mathrm{d}\mathbf{x}_d \tag{S48}$$

Supplementary References

[1] Sheppard, C. J. R., Mehta, S. B. & Heintzmann, R. Superresolution by image scanning microscopy using pixel reassignment. *Optics Letters* **38**, 2889 (2013).

[2] De Luca, G. M. *et al.* Re-scan confocal microscopy: scanning twice for better resolution. *Biomed. Opt. Express* **4**, 2644–2656 (2013).

[3] Roth, S., Sheppard, C. J. R., Wicker, K. & Heintzmann, R. Optical photon reassignment microscopy (opra). *Opt. Nanoscopy* **2**, 5 (2013).

[4] York, A. G. *et al.* Instant super-resolution imaging in live cells and embryos via analog image processing. *Nat. Methods* **10**, 1122–1126 (2013).

[5] Azuma, T. & Kei, T. Super-resolution spinning-disk confocal microscopy using optical photon reassignment. *Opt. Express* **23**, 15003–15011 (2015).

[6] York, A. G. *et al.* Resolution doubling in live, multicellular organisms via multifocal structured illumination microscopy. *Nat. Methods* **9**, 749–754 (2012).

[7] Schulz, O. *et al.* Resolution doubling in fluorescence microscopy with confocal spinning-disk image scanning microscopy. *Proc. Natl. Acad. Sci. U. S. A.* **110**, 21000–21005 (2013).

[8] Vicidomini, G., Moneron, G., Eggeling, C., Rittweger, E. & Hell, S. W. STED with wavelengths closer to the emission maximum. *Opt. Express* **20**, 5225–5236 (2012).

[9] Tortarolo, G., Castello, M., Koho, S. & Vicidomini, G. Synergic combination of stimulated emission depletion microscopy with image scanning microscopy to reduce light dosage. *bioRxiv* (2019).

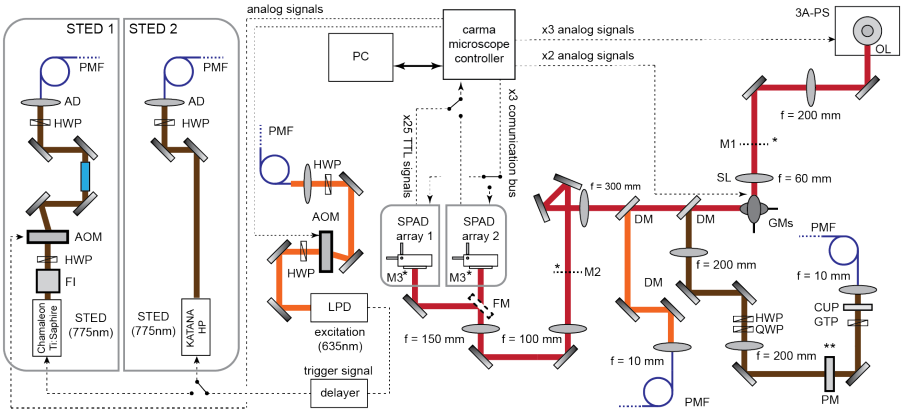

Figure S1: **The STED-ISM setup.** HWP:half-waveplate;AOM:acousto-optic modulator; AD:achromatic doublet; PMF: polarized-maintaining fiber; FI: Faraday isolator; GR: glass-road; 3A-PS: three-axis piezo stage; SL: scanning lens; GNs: galvanometric mirrors; DM: dichroic mirror; FM: flip mirror; QWP: quarter-wave plate; BPF: band pass filter; MMF: multi-modes fiber; APD: avalanche photo-diode; PM: phase-mask; GTP: Glan-Thompson polarizer; CUP: clean-up filter. Magnifications: M1 = 60×, M2 = 300×, M3 = 450×. The asterisk denotes the plane conjugate to the image plane. The double asterisks denote the plane conjugate to the objective back-aperture.

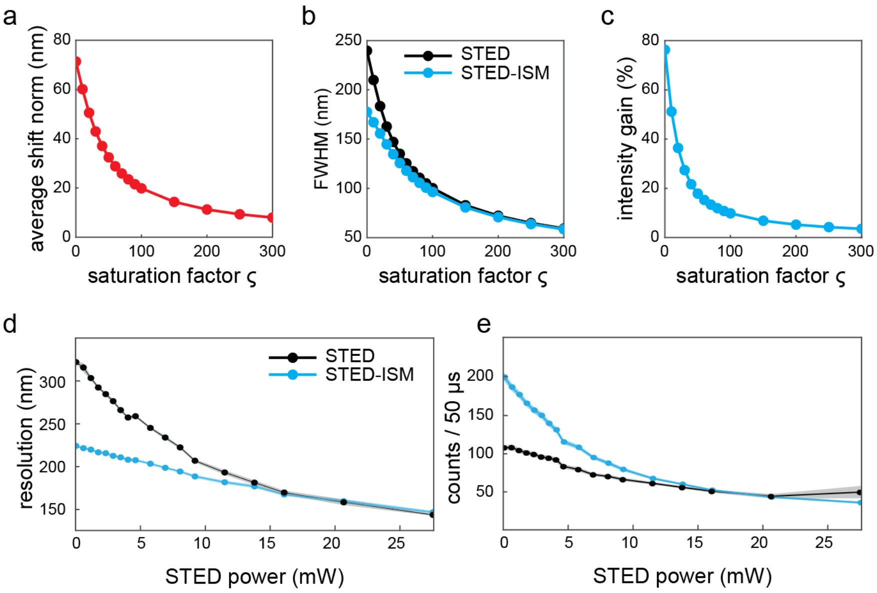

Figure S2: **STED-ISM benefits.** In **a**, we calculate the average norm of the shift vectors as a function of the saturation factor (ς). For increasing STED power, the images are less shifted. In **b**, we compare the resolution (calculated as the FWHM of the PSF) of raw STED versus STED-ISM. In **c**, we present the reduction of beam power that we can apply to maintain the same lateral resolution of raw STED. The benefit of STED-ISM is maximal at low saturation factors. In **d**, we show the experimental measure of the resolution of the images of the fluorescent beads of Fig. 3a before (black curve) and after (blue curve) adaptive pixel reassignment. The resolution is measured as the FWHM of the result of a fit of the images of the beads to a Gaussian function. The graph reports the average values and the corresponding standard error. In **e**, we show the experimental measure of the signal of the image of the fluorescent beads of Fig. 3a before (black curve) and after (blue curve) adaptive pixel reassignment. The signal is measured as the peak value of the images of the beads. The graph reports the average values and the corresponding standard error. Note that at high depletion power the SNR of the raw dataset decreases, potentially hindering the successful ISM reconstruction. Thus, the peak signal level of the ISM images could appear lower than that of the original images.

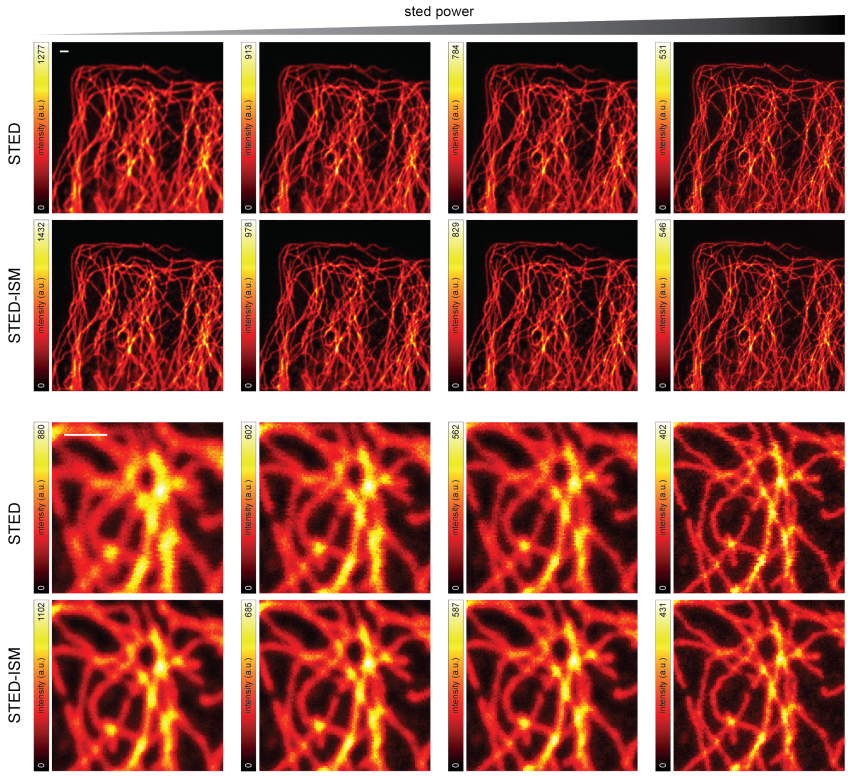

Figure S3: **STED-ISM measurements of fixed cells with increasing STED power**. The resolution and the SNR of are enhanced by STED-ISM when imaging tubulin-labelled fixed Hela cells. Retrieved FRC resolution values: 278 and 189 nm, 202 and 180 nm, 192 and 177 nm, 136 and 136 nm, for raw STED and STED-ISM, respectively. Dwell time: 100 µs; pixel size: 40 nm; STED powers: 0, 15, 23, 137 mW; format: 500 × 500 pixels; details format: 150 x 150 pixels; all scale-bars: 1 µm.

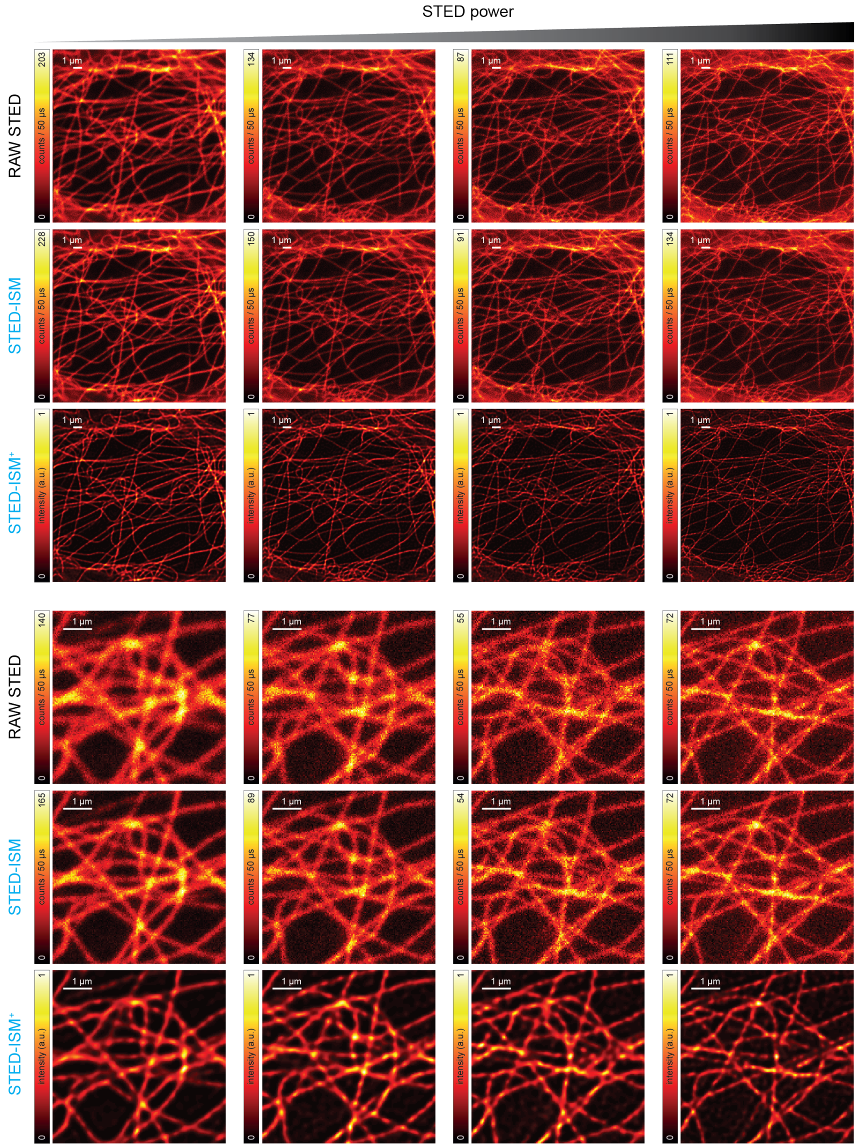

Figure S4: **Live cell STED-ISM imaging with increasing STED power**. Retrieved FRC resolution values: 290 and 213 nm, 233 and 202 nm, 202 and 202 nm, 168 and 165 nm, for raw STED and STED-ISM, respectively. Pixel dwell time: 50 µs. Pixel size: 40 nm. STED powers: 0, 10, 20, 45 mW. Format of the full images: 500 × 500 pixels. Format of the details: 150 × 150 pixels. All scale-bars: 1 µm

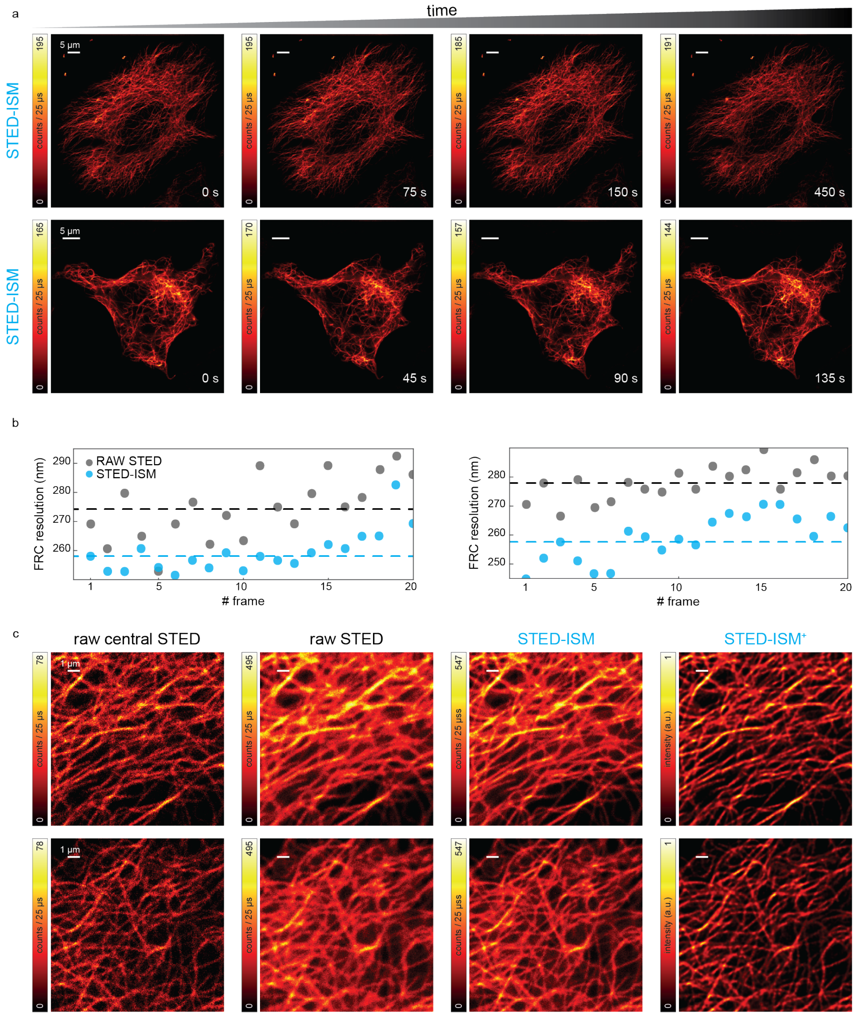

Figure S5: **STED-ISM time series.** **a**, Two extended time series (20 frames, 500 and 300 s) of Sir-Tubulin labeled Hela cells. Different frames are reported to show the negligible photo-bleaching. **b**, The FRC analysis confirms, for both time series, the superior performance of STED-ISM compared to raw STED. **c**, Details from the time series, showing the image recorded by the central element of the SPAD array (raw central STED), the result of the summing operation (raw STED), the result of the pixel reassignment operation (STED-ISM), and the deconvolution image (STED-ISM$^+$). Pixel dwell time: 25 µs. Pixel size: 70 and 67 nm. Format of the full images: 1000×1000 and 750×750 pixels. Format of the details: 200×200 pixels. 635 nm excitation laser power: 8 µW; 775 nm STED laser power: 21 mW. Scale bars of **a**: 5 µm. Scale bars of **c**: 1 µm.

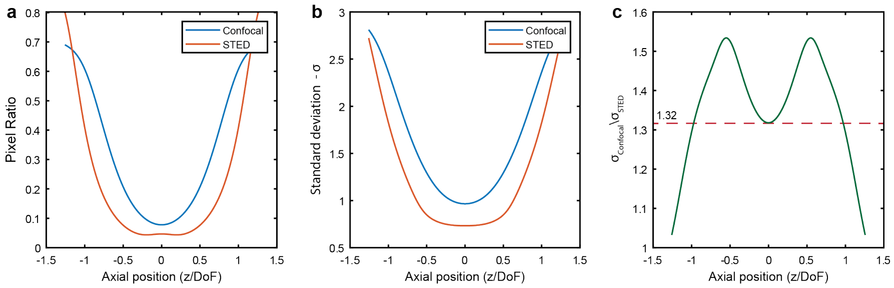

Figure S6: **Distribution of light on the SPAD array.** From the PSF simulations presented in Figure 4 we calculated a z-stack of fingerprints. In **a**, we show the ratio of the intensity averaged over the border elements to the intensity of the central element of the fingerprint. In **b**, we show the standard deviation resulting from a Gaussian fit to the fingerprints. In **c**, we show the ratio of the standard deviation of confocal fingerprint to the stadard deviation of the STED fingerprint. In the focal plane, the ratio is 1.32 to be compared with the theoretical expected value of 1.38. The axial units are normalized with respect to the depth-of-field (DoF = $\lambda n/\mathrm{NA}^2$).

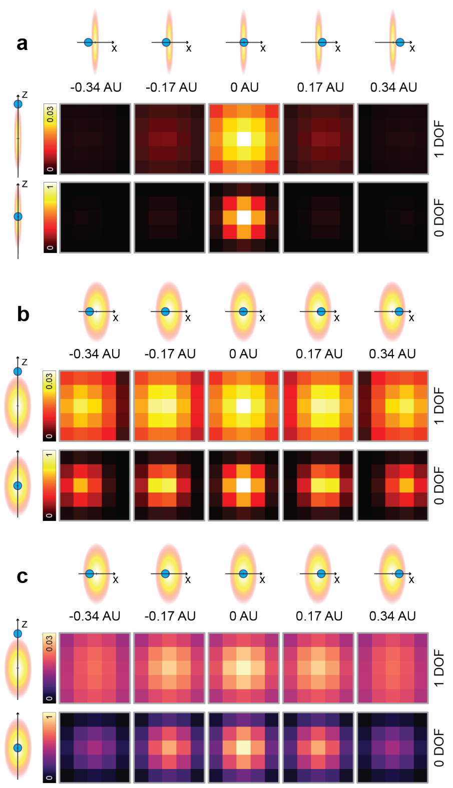

Figure S7: **STED and confocal micro-images.** Simulated micro-images generated by a single-emitter at different lateral and axial positions. In **a** we imposed ideal STED conditions ($\varsigma = 300$). The micro-image corresponds to the detection PSF and contains a non-negligible amount of signal only if the emitter is centered in the xy plane. The lateral confinement occurs also out-of-focus. In **b**, we simulated the confocal case. The micro-images shift as the emitter moves laterally. In **c**, we show the micro-images from **b** after adaptive pixel reassignment. The new micro-images are now all centred and independent from the sample structure, except for a scale factor. In detail, they are proportional to the fingerprint of the corresponding axial plane. For visualization purposes, the simulatons are run with a detector size of one Airy unit.

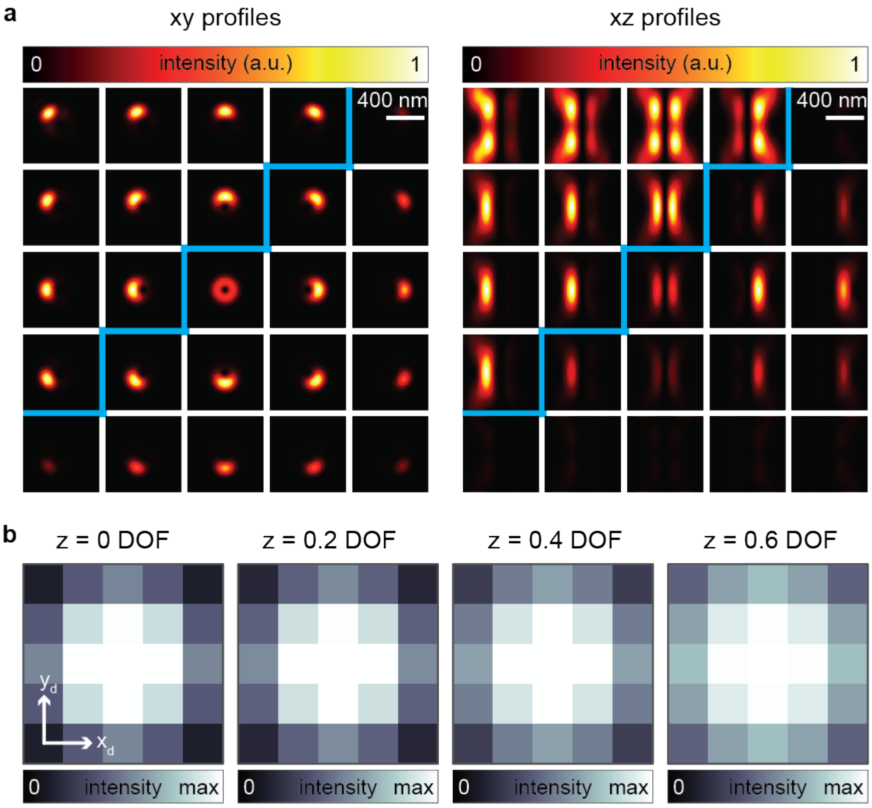

Figure S8: **PSF in case of annular excitation.** In **a**, we show the confocal PSFs obtained using an annular illumination. We calculated the PSFs for each detector element and for both the lateral and the axial plane. In **b**, we show the fingerprints calculated by summing all the scan points of the sections of the PSF at different axial positions. Notably, the intensity profile is flatter than in the case of Gaussian beam excitation.

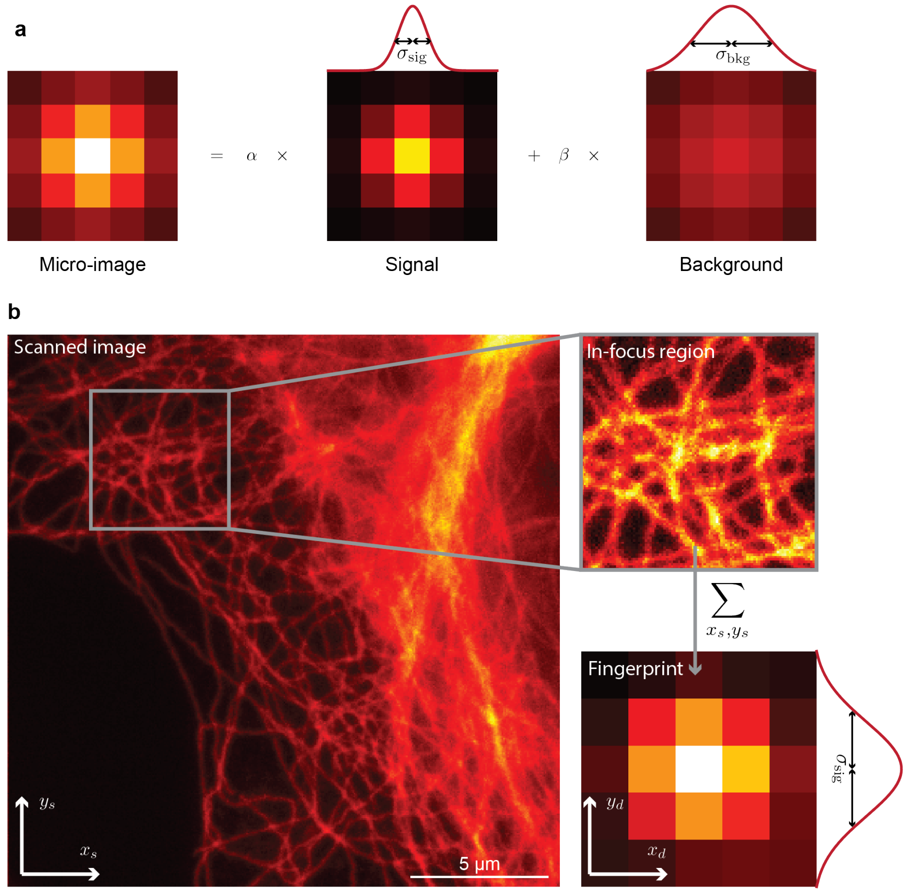

Figure S9: **Working principle of the Focus-STED algorithm.** In **a**, we show the core of the analysis: each micro-image is fitted to two Gaussian functions. The micro-image is normalized before the fitting and the weights respect the condition $\alpha + \beta = 1$. The width of the in-focus Gaussian function is fixed either by using the theoretical value or by an additional measurement. In **b**, we show how to experimentally measure σ_{sig}. We select a sub-image containing only in-focus emitters and calculate the corresponding fingerprint. The result is fitted to a single Gaussian function, whose standard deviation is used as σ_{sig} for the pixel-by-pixel fitting of the model presented in **a**.

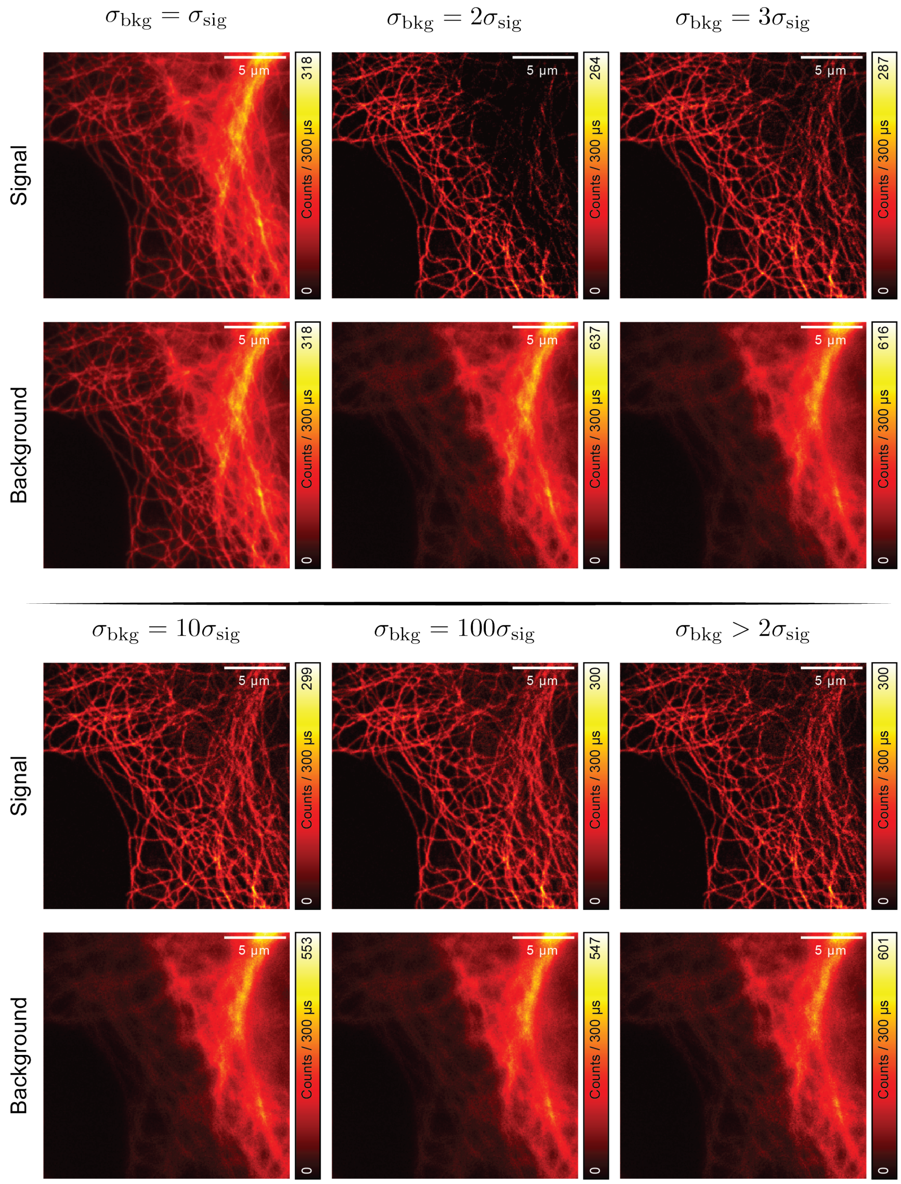

Figure S10: **Result of the Focus-ISM algorithm.** The first five reconstructions are obtained by fixing $\sigma_{\rm bkg}$. For $\sigma_{\rm bkg} = \sigma_{\rm sig}$ the in-focus and out-of-focus photons follow exactly the same distribution and cannot be distinguished by the algorithm. Thus, the light is simply split into two identical images. For increasing $\sigma_{\rm bkg}$, the algorithm distinguishes the two contributions, but if $\sigma_{\rm bkg}$ is too small some misclassification may happen and too much signal is removed. For divergent $\sigma_{\rm bkg}$, the shape of the background approaches a constant offset. Notably, the result is still superior of that of the simple subtraction because of the physical constraint imposed by the fitting process. The last reconstruction is obtained letting $\sigma_{\rm bkg}$ be a fitting parameter, with the only physical constraint of being much larger than $\sigma_{\rm sig}$. With this last approach, $\sigma_{\rm bkg}$ is found adaptively pixel-by-pixel.

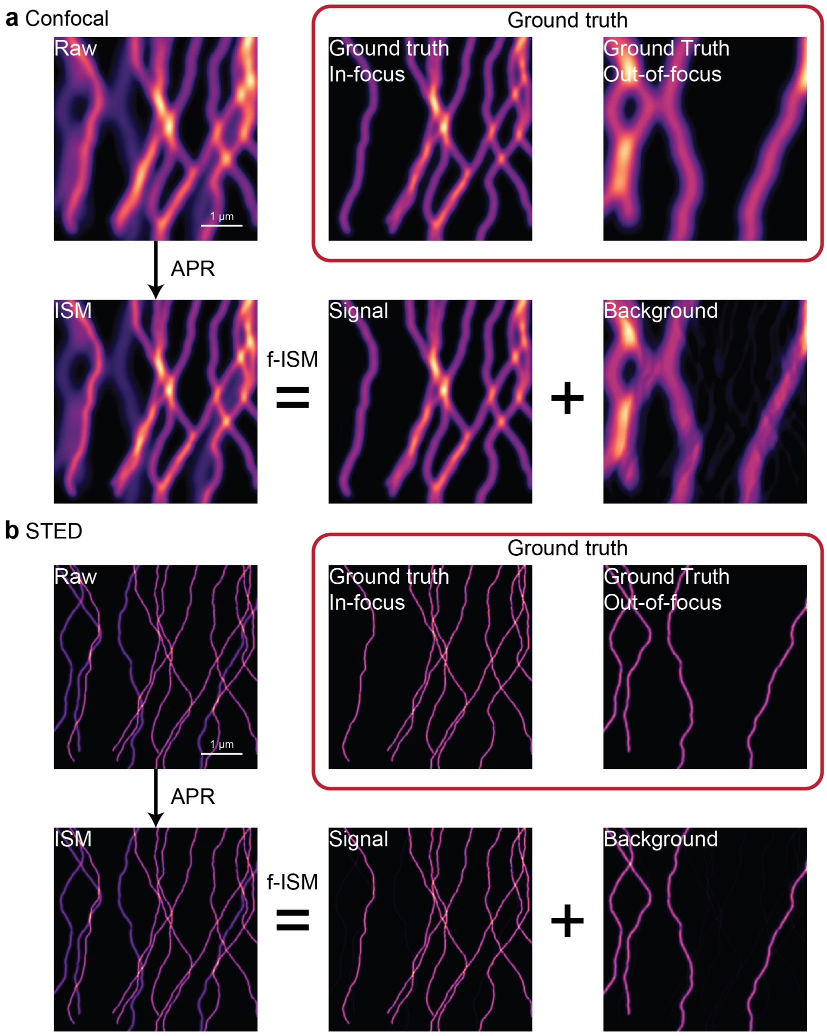

Figure S11: **Result of Focus-ISM compared to the ground-truth.** For each panel, we simulated tubulin filaments in-focus ($z = 0$) and out-of-focus ($z = 1\,\text{DoF}$). We summed the two planes, generating an image affected by background (*raw*). Then, we performed adaptive pixel reassignment, generating the ISM image. Lastly, we analyzed with the f^2-ISM algorithm each micro-image of the reassigned dataset. The result is two images, one containing only the in-focus component (*signal*) and one containing only the out-of-focus component (*background*). On the bottom, we show the *ground-truth* for each component. Both in the confocal (**a**) and STED case (**b**) the result is very close to the corresponding ground-truth.

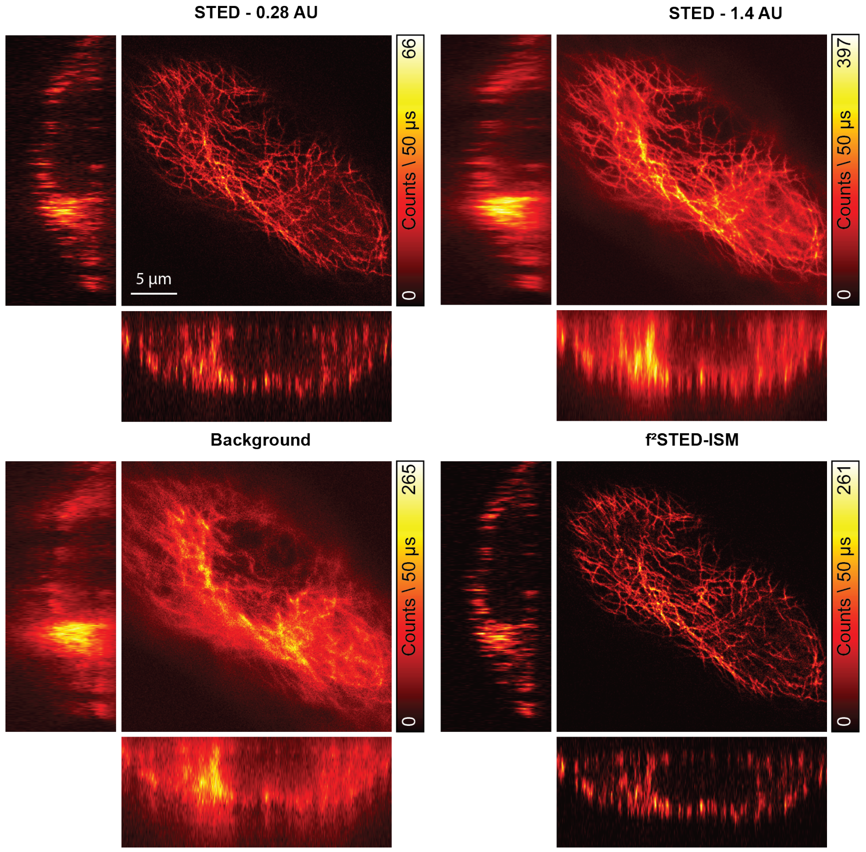

Figure S12: **Focus-ISM applied to a z-stack of STED images. Top left**, STED image acquired with a closed pinhole. The SBR is high, but the SNR is low. **Top right**, STED image acquired with an open pinhole. The SNR is high, but the SBR is low. **Bottom right**, result of the focus-ISM algorithm applied to the same dataset of the above images. Both SNR and SBR are high, and the similarity with the closed-pinhole image proves the absence of artifacts. **Bottom left**, image of the background removed. The xz and yz slices are taken from the middle of the volume and are linearly interpolated along the z-axis for visualization purposes. All images were acquired using an STED power of 125 mW measured before the objective lens.

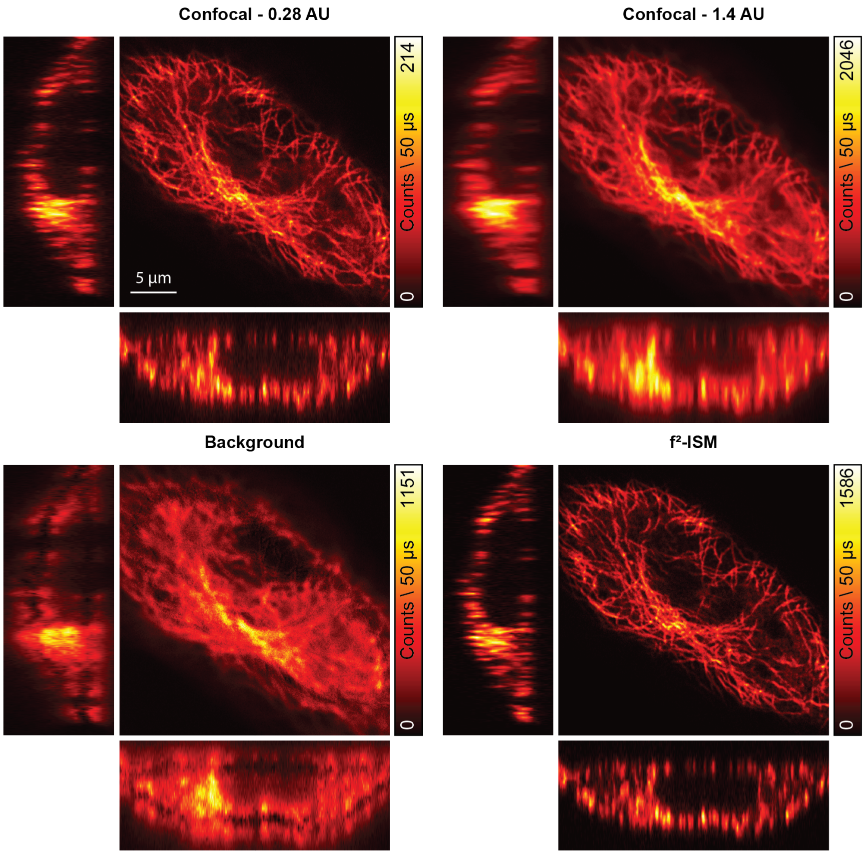

Figure S13: **Focus-ISM applied to a z-stack of confocal images. Top left**, confocal image acquired with a closed pinhole. The SBR is high, but the SNR is low. **Top right**, STED image acquired with an open pinhole. The SNR is high, but the SBR is low. **Bottom right**, result of the focus-ISM algorithm applied to the same dataset of the above images. Both the SNR and SBR are high, and the similarity with the closed-pinhole image proves the absence of artifacts. **Bottom left**, image of the background removed. The xz and yz slices are taken from the middle of the volume and are linearly interpolated along the z-axis for visualization purposes.

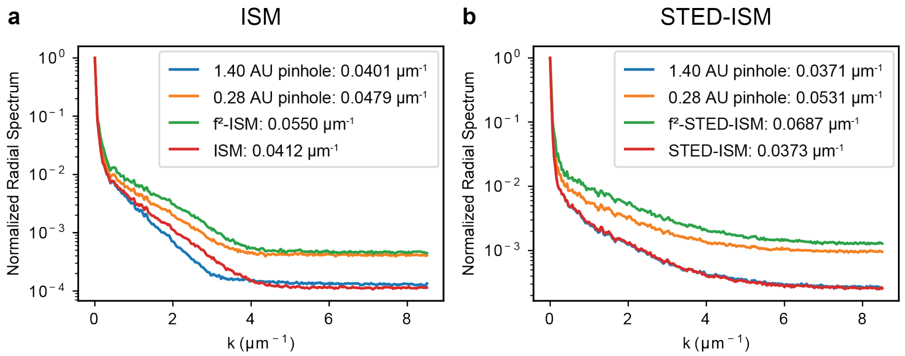

Figure S14: **Radial spectra of experimental images**. The spectra have been obtained by calculating the Fourier transform of the XY images shown in Figures 6, S12, and S13, performing the angular average, and normalizing the result with respect to the mean of the image. The legend contains the value of the integral of the spectra over the spatial frequencies k. A higher value of the integral can result from an increased spatial resolution – namely if the cut-off frequency is higher – or if the contrast at high frequencies is enhanced with respect to the low frequencies. **a**, the superior lateral resolution of ISM, compared to LSM (1.40 AU), determines a larger a cut-off frequency and a larger integral value. Although having poor SNR, LSM (0.28 AU) has superior lateral resolution and greater optical sectioning, thus showing higher contrast of large spatial frequencies and a bigger integral value. Focus-ISM has good SNR, the same lateral resolution of ISM, and the best optical sectioning capability. These features result in the biggest integral value. **b**, STED (1.40 AU) and STED-ISM have a comparable radial spectrum, due to the high power of the depletion beam. STED (0.28 AU) has better high-frequency contrast, but the best contrast is still achieved by Focus-STED-ISM.

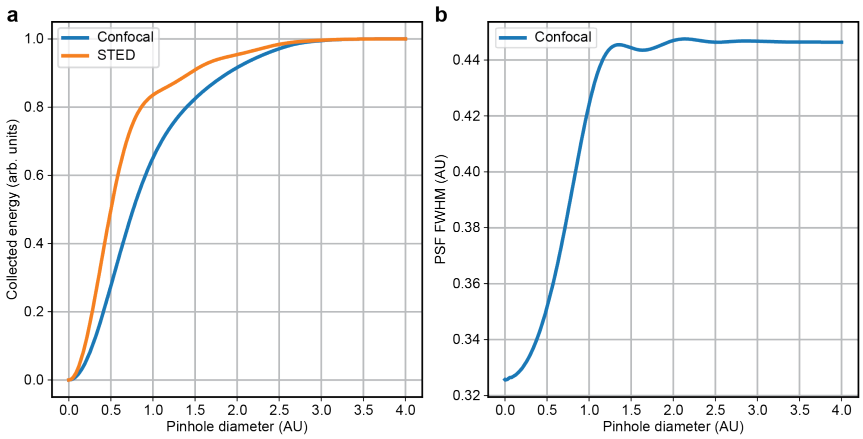

Figure S15: **Collection efficiency at different pinhole sizes.** In **a**, we show the results of the simulated collection efficiency from an in-focus sample at different pinhole size. In the STED case, the distribution of the light on the detector plane is given by the detection PSF. In the confocal case the light distribution is given by the convolution of the excitation PSF with the detection PSF, thus broader of a factor $\sqrt{2}$ at the focal plane. Thus, the correct estimation of the correct pinhole size should consider the full fingerprint. The detection PSF is a an underestimation of intensity distribution on the detector, except than in the ideal STED case. In **b**, we show the full width at half maximum (FWHM) of the confocal PSF as a function of the diameter of the pinhole. The resolution and the collected signal are inversely proportional.

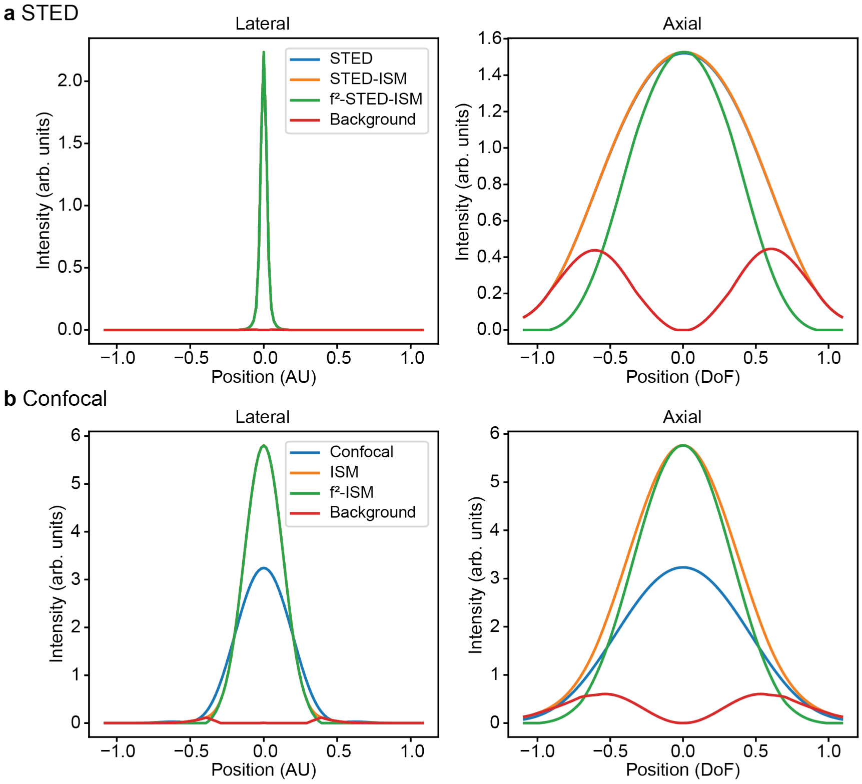

Figure S16: **Intensity profiles of the reconstructed PSF.** Intensity profiles of the PSFs shown in Fig. 4. Each line is extracted from the corresponding axis of symmetry of the PSF, namely $z = 0$ for the lateral profile and $x = 0$ for the lateral profile. In **a** we show the profiles of the STED PSFs ($\varsigma = 300$), and in **b** we show the profiles of the confocal PSFs.

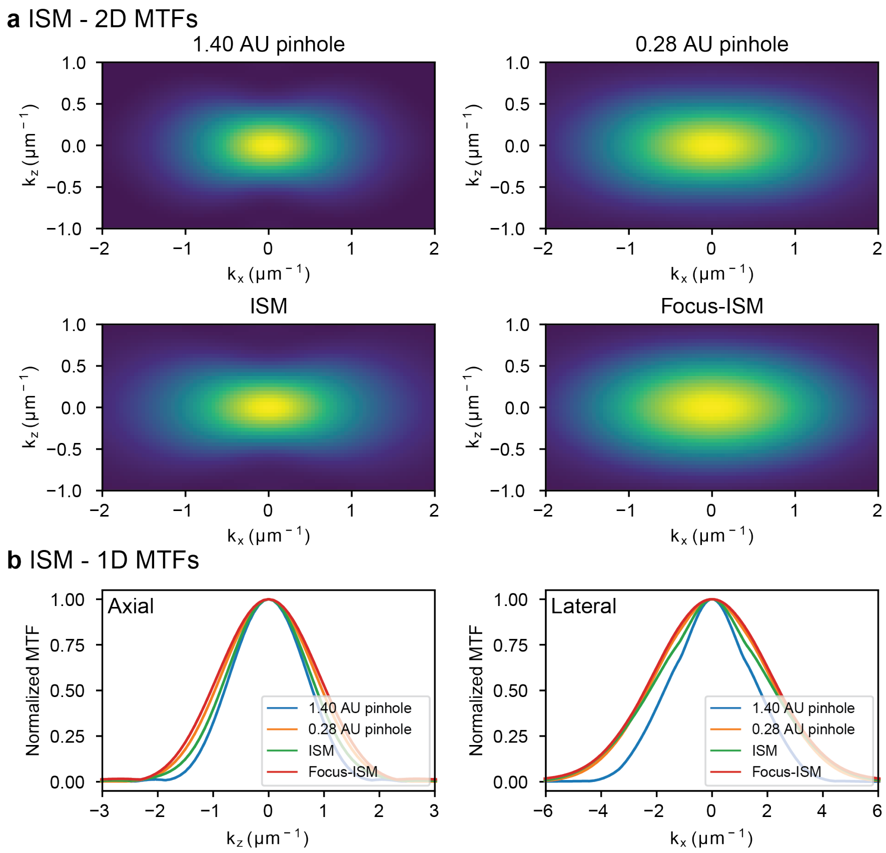

Figure S17: **ISM Modulation transfer functions**. Simulated MTFs, corresponding to the PSFs shown in Fig. 4. **a**, 2D MTFs of LSM (pinhole size: 1.40 and 0.28 AU), ISM and focus-ISM, plotted over the axial (k_z) and lateral (k_x) spatial frequencies. The MTF of LSM with an open pinhole (1.40 AU) and the MTF of ISM differ only in the extension along the k_x-axis, due to the improved lateral resolution of ISM, but they both show the *missing cone* along the k_z-axis, namely the lack of optical sectioning. The MTF of LSM with a closed pinhole (0.28 AU) and of focus-ISM present the same extension along the k_x-axis, and a similar extension along the k_z-axis, without the presence of the *missing cone*. **b**, 1D Normalized MTFs, obtained by integrating the corresponding 2D MTFs of **a** along the axial or lateral dimension. The axial profile clearly shows the superior optical sectioning of focus-ISM compared to LSM (0.28 AU), while keeping a signal content comparable to that of LSM (1.40 AU). The lateral profile shows the enhanced lateral resolution of ISM and focus-ISM with respect to LSM (1.40 AU).

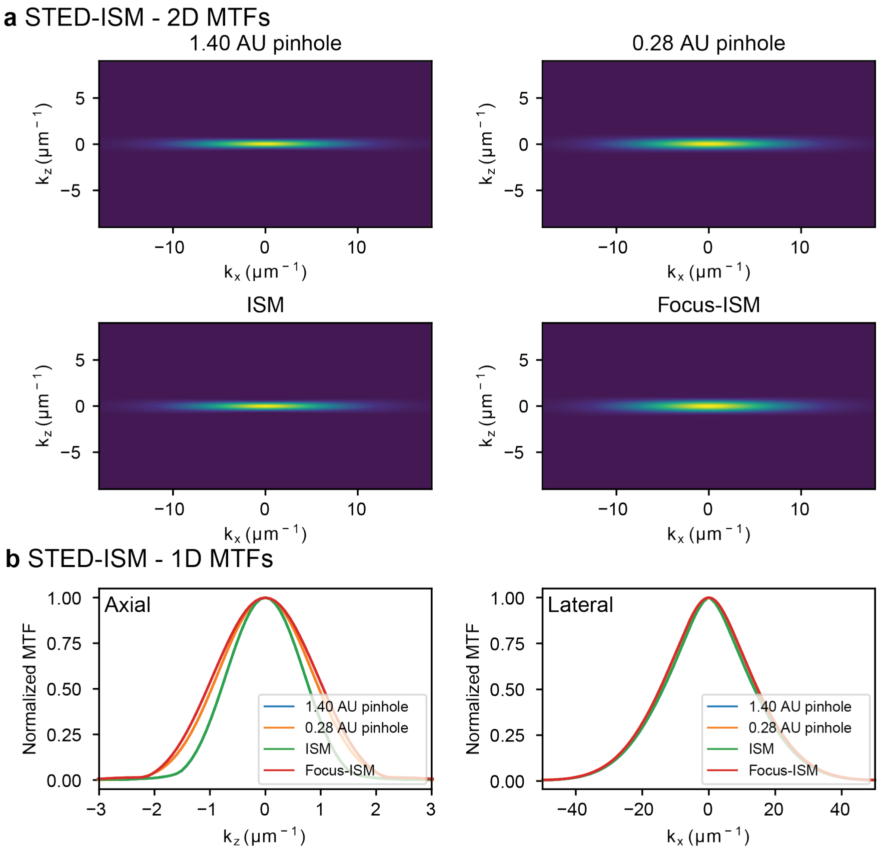

Figure S18: **STED-ISM Modulation transfer functions**. Simulated MTFs, corresponding to the PSFs shown in Fig. 4. **a**, 2D MTFs of STED (pinhole size: 1.40 and 0.28 AU), STED-ISM and focus-STED-ISM at saturation factor $\varsigma = 300$. **b**, 1D Normalized MTFs, obtained by integrating the corresponding 2D MTFs of **a** along the axial or lateral dimension. In this case all the lateral MTFs have the same support on the k_x-axis because of the high-value of ς. The axial MTFs show that Focus-STED-ISM still improves the optical sectioning, as shown by the higher contrast of the axial MTF compared to the others.
