# Supplementary material for: Focus image scanning microscopy for sharp and gentle super-resolved microscopy
Source: Nat Commun. 2022 Dec 13;13:7723. doi: 10.1038/s41467-022-35333-y (PMC9747786; doi:10.1038/s41467-022-35333-y)
Supplement: Supplementary file 3 — Reporting Summary [file 41467_2022_35333_MOESM3_ESM.pdf]

nature portfolio

Corresponding author(s): Dr. Giuseppe Vicidomini

Last updated by author(s): Nov 8, 2022

Reporting Summary

Nature Portfolio wishes to improve the reproducibility of the work that we publish. This form provides structure for consistency and transparency in reporting. For further information on Nature Portfolio policies, see our Editorial Policies and the Editorial Policy Checklist.

Statistics

For all statistical analyses, confirm that the following items are present in the figure legend, table legend, main text, or Methods section.

n/a	Confirmed	
☒	☐	The exact sample size (*n*) for each experimental group/condition, given as a discrete number and unit of measurement
☒	☐	A statement on whether measurements were taken from distinct samples or whether the same sample was measured repeatedly
☒	☐	The statistical test(s) used AND whether they are one- or two-sided<br>*Only common tests should be described solely by name; describe more complex techniques in the Methods section.*
☒	☐	A description of all covariates tested
☒	☐	A description of any assumptions or corrections, such as tests of normality and adjustment for multiple comparisons
☐	☒	A full description of the statistical parameters including central tendency (e.g. means) or other basic estimates (e.g. regression coefficient) AND variation (e.g. standard deviation) or associated estimates of uncertainty (e.g. confidence intervals)
☒	☐	For null hypothesis testing, the test statistic (e.g. *F*, *t*, *r*) with confidence intervals, effect sizes, degrees of freedom and *P* value noted<br>*Give P values as exact values whenever suitable.*
☒	☐	For Bayesian analysis, information on the choice of priors and Markov chain Monte Carlo settings
☒	☐	For hierarchical and complex designs, identification of the appropriate level for tests and full reporting of outcomes
☒	☐	Estimates of effect sizes (e.g. Cohen's *d*, Pearson's *r*), indicating how they were calculated

Our web collection on statistics for biologists contains articles on many of the points above.

Software and code

Policy information about availability of computer code

Data collection	All microscopy images have been collected using our custom data acquisition/visualization/processing software, which is also in charge to control the synchronization between the galvanometric mirrors, and the SPAD array detector.
Data analysis	Image analysis (adaptive pixel reassignment and Focus-ISM) has been performed using custom python algorithms, publicly available at: https://github.com/Alessandro-Zunino/Focus-ISM. The simulations have been performed using the code from Leutenegger et al., that can be found at https://documents.epfl.ch/users/l/le/leuteneg/www/FocusFields/index.html

For manuscripts utilizing custom algorithms or software that are central to the research but not yet described in published literature, software must be made available to editors and reviewers. We strongly encourage code deposition in a community repository (e.g. GitHub). See the Nature Portfolio guidelines for submitting code & software for further information.

Data

Policy information about availability of data

All manuscripts must include a data availability statement. This statement should provide the following information, where applicable:

- Accession codes, unique identifiers, or web links for publicly available datasets
- A description of any restrictions on data availability
- For clinical datasets or third party data, please ensure that the statement adheres to our policy

The experimental data generated and analysed in this study have been deposited in the Zenodo database and can accessed at https://doi.org/10.5281/zenodo.7303679.

Human research participants

Policy information about studies involving human research participants and Sex and Gender in Research.

Reporting on sex and gender	N/A
Population characteristics	N/A
Recruitment	N/A
Ethics oversight	N/A

Note that full information on the approval of the study protocol must also be provided in the manuscript.

Field-specific reporting

Please select the one below that is the best fit for your research. If you are not sure, read the appropriate sections before making your selection.

☒ Life sciences ☐ Behavioural & social sciences ☐ Ecological, evolutionary & environmental sciences

For a reference copy of the document with all sections, see nature.com/documents/nr-reporting-summary-flat.pdf

Life sciences study design

All studies must disclose on these points even when the disclosure is negative.

Sample size	The results of our method demonstrate the capabilities to obtain high resolution and high SNR STED-ISM images and to remove the out-of-focus light from ISM images. As such, the results do not depend on the statistical variations or the properties of the used samples and we performed the analysis on individual images. The only dataset for which the sample size was relevant is that corresponding to Figure 3a-b. In that case we chose a field of view large enough to contain roughly 50 beads, which are more than enough to estimate the average resolution of the optical system.
Data exclusions	No data were excluded from the analyses.
Replication	The data of Figure 3a, 3b, and S2d has been acquired once, but the image has been acquired with a field of view large enough to contain around 50 beads to add statistical significance to the single-bead analysis.<br>The results of Figure 3d, 3e, S3, S4, S5 have been reproduced on similar samples ten times.<br>The results of Figure 5, S9, and S10 have been reproduced on similar samples five times.<br>The results of Figure 6, S12, S13, and S14 have been reproduced on the full stack of 31 planes.
Randomization	This is not relevant for the method, because it does not depend on the statistical variation of the properties of the samples
Blinding	This is not relevant for the method, because it does not depend on the statistical variation of the properties of the samples.

Behavioural & social sciences study design

All studies must disclose on these points even when the disclosure is negative.

Study description	N/A

Research sample	N/A
Sampling strategy	N/A
Data collection	N/A
Timing	N/A
Data exclusions	N/A
Non-participation	N/A
Randomization	N/A

Ecological, evolutionary & environmental sciences study design

All studies must disclose on these points even when the disclosure is negative.

Study description	N/A
Research sample	N/A
Sampling strategy	N/A
Data collection	N/A
Timing and spatial scale	N/A
Data exclusions	N/A
Reproducibility	N/A
Randomization	N/A
Blinding	N/A

Did the study involve field work? ☐ Yes ☒ No

Reporting for specific materials, systems and methods

We require information from authors about some types of materials, experimental systems and methods used in many studies. Here, indicate whether each material, system or method listed is relevant to your study. If you are not sure if a list item applies to your research, read the appropriate section before selecting a response.

Materials & experimental systems

n/a	Involved in the study	
☐	☒	Antibodies
☐	☒	Eukaryotic cell lines
☒	☐	Palaeontology and archaeology
☒	☐	Animals and other organisms
☒	☐	Clinical data
☒	☐	Dual use research of concern

Methods

n/a	Involved in the study	
☒	☐	ChIP-seq
☒	☐	Flow cytometry
☒	☐	MRI-based neuroimaging

Antibodies

Antibodies used	Anti-a-Tubulin antiserum, Sigma-Aldrich, T5168, clone B-5-1-2, dilution 1:800.<br>Abberior Star red Goat anti-mouse IgG, Abberior, 2-0002-011-2, dilution 1:1000
Validation	Provided by the suppliers:<br>https://www.sigmaaldrich.com/IT/it/product/sigma/t5168<br>https://www.sigmaaldrich.com/IT/it/product/sigma/52283

Eukaryotic cell lines

Policy information about cell lines and Sex and Gender in Research

Cell line source(s)	HeLa, sigma, 93021013
Authentication	None of the cells line used were authenticated.
Mycoplasma contamination	Cell lines were not tested for contaminations.
Commonly misidentified lines (See ICLAC register)	No commonly misidentified cell line were used
